# Role of Environmental Photocatalysts and Organic Matter on the Degradation and Toxicity of Metformin Hydrochloride

**DOI:** 10.3390/toxics13050407

**Published:** 2025-05-17

**Authors:** Rifat Khan, Jaqueline Regalado, Malsha Indeewari Kanaththage, Praveen L. Patidar, Gayan Rubasinghege

**Affiliations:** Department of Chemistry, New Mexico Institute of Mining & Technology, 801 Leroy Place, Socorro, NM 87801, USA; rifat.khan@student.nmt.edu (R.K.); jaqueline.regalado@student.nmt.edu (J.R.); malshaindeewari.kanaththage@student.nmt.edu (M.I.K.); praveen.patidar@nmt.edu (P.L.P.)

**Keywords:** metformin hydrochloride, photodegradation, degradation products, pharmaceuticals, ecotoxicity

## Abstract

Metformin is the preferred first-line treatment for non-insulin-dependent diabetes mellitus, known for its benefits in cancer suppression, weight loss, and antiketogenic activity. It is a leading drug regarding mass distribution, and its high solubility in water leads to its significant accumulation in surface and groundwater. While some studies have explored its degradation products and toxicological consequences, none have specifically examined the impact of individual natural minerals and their mechanisms leading to these degraded compounds. Our investigation focuses on understanding the mineralogical effects of different photocatalysts and organic matter while assessing acute toxicity through cell viability tests on human cell lines. We utilized a custom-built reactor system containing metformin hydrochloride, photocatalysts, and organic matter under oxidizing conditions to explore the formation of new degraded compounds. We assessed the acute toxicity of both metformin hydrochloride and the resulting chemical mixture on kidney and liver cell lines using the colorimetric MTT cell viability assay. Despite the abundance of surface functional groups in organic humic acid, only solar energy-driven catalysts were found to effectively break down this widely used medication. Comparative analysis of metformin hydrochloride and its degraded residues indicates a toxic effect on liver cells. Our experiments contribute to understanding the environmental fate of metformin and pave the way for further biochemical investigations to identify toxicological mechanisms.

## 1. Introduction

The rise in human activities has led to numerous environmental challenges, particularly impacting aquatic ecosystems. Various emerging contaminants increasingly pollute these environments, yet the associated risks to public health and the environment largely remain unassessed [1]. Pharmaceuticals intended for both human and veterinary use are a significant concern due to their diverse structures and chemical properties. While wastewater treatment plants do process industrial pharmaceutical waste, these treatments are often inadequate to eliminate the harmful effects of these substances on the environment. Additionally, the growing use of pharmaceuticals and their disposal as household waste further exacerbates the problem.

Metformin hydrochloride (1,1-dimethylbiguanide hydrochloride) is a US FDA-approved oral hypoglycemic drug recommended as the first-line therapy for patients with type II diabetes. Often referred to as a “wonder drug”, metformin is celebrated for its additional cardiovascular benefits and favorable safety profile [2]. In addition to its primary use, metformin is also prescribed off-label for conditions such as prediabetes, gestational diabetes, polycystic ovary syndrome (PCOS), and weight loss, making it one of the most widely prescribed medications globally. As a basic, hydrophilic drug, metformin exists as a cation at physiological pH [3]. It helps restore the body’s insulin response, thereby aiding in the reduction of blood sugar levels [4]. Notably, the human body does not metabolize metformin, which is excreted unchanged through the kidneys. This raises concerns about its potential environmental impact, as it can accumulate in waterways through human waste. Metformin exhibits high polarity, high water mobility, and a low octanol/water partition coefficient [5]. Its concentrations vary from micrograms per liter (µg/L) to nanograms per liter (ng/L) in different environments. Consequently, metformin can concentrate in the ecosystem, posing a significant threat to human health, wildlife, and aquatic organisms.

Metformin is a micromolecule, and its structure is hygroscopic in nature [6]. It contains two joined guanidine (biguanide) moieties along with two imine groups (C=N–H), as well as primary, secondary, and tertiary amine functional groups [7]. These groups make metformin hydrophilic and influence its chemical reactivity. The breakage of C-N bonds in the structure produces guanylurea along with dimethylamine [1C]. Studies have shown that strong oxidants (e.g., hydroxyl radicals) readily attack the biguanide linkage and yield smaller molecules like methylbiguanide and even ring-generating products [8].

The widespread presence of metformin in the environment poses significant risks to various species. For example, endocrine-disrupting effects have been evaluated at environmentally relevant concentrations on adult *Pimephales promelas* (fathead minnows). This exposure significantly increased the expression of vitellogenin mRNA in male fish, suggesting potential endocrine disruption [9]. In *Pelophylax nigromaculatus* tadpoles, exposure to metformin altered the intestinal microbiota composition. Pathogenic bacterial genera such as *Salmonella*, *Comamonas*, and *Stenotrophomonas* were found to increase, while beneficial genera like *Blautia* and *Prevotella* decreased [10]. Additionally, the uptake and accumulation of metformin and its degradation products have been observed in cereal grains, barley, and potato peels [11]. Given these reports on its environmental persistence and biological impacts, metformin is recognized as a pharmaceutical of significant ecological concern. Like many pharmaceuticals, current wastewater treatment technologies are inadequate in entirely removing metformin from wastewater streams, resulting in substantial quantities of the compound entering aquatic ecosystems. The saying “Everything in excess is opposed to nature” is especially relevant in the context of metformin today.

Numerous studies have been conducted to evaluate the degradation of metformin. Luo et al. investigated the transformation of metformin under acidic, basic, oxidative, and accelerated storage conditions [12]. Their work reported that while metformin remained stable in basic conditions, it degraded under acidic, oxidative, and accelerated storage environments. This study further identified four impurities, with 1,4-dihydro-4-imino-1-methyl-1,3,5-triazin-2-amine being the most abundant and newly reported impurity. Carbuloni et al. conducted experiments using the photocatalyst TiO_2_–ZrO_2_ to observe the degradation of metformin. Their findings indicated that this photocatalyst could degrade 50% of metformin after just 30 min of UV irradiation [13]. The fate and transformation of metformin and its degradation products in wastewater treatment plants (WWTPs) and surface waters have also been investigated [14]. Notably, some degradation products were found in more significant quantities in treated effluent than in the influent, even after treatment processes. Photolysis, photocatalysis, ozonation, and chlorination have proven very effective in treating metformin and its degradation products in wastewater [15]. In addition to examining degradation, some studies have looked into the cytotoxic effects of metformin and its degradation products on fibroblast cells. These studies indicated that cell viability ranged from 85% to 100%, suggesting low acute toxicity [16]. Although significant research has been conducted on metformin and its degradation products, none has specifically focused on the effects of individual environmental minerals on metformin.

Metformin can undergo various environmental abiotic degradation pathways when exposed to sunlight, water, and minerals. These processes lead to the formation of numerous degradation products from the parent compound. However, our understanding of the degradation mechanisms of metformin and the identities of its degradation products in environmental settings remains limited. Elucidating these pathways is essential for predicting the transformation and potential health impacts of metformin and its byproducts. The current work focuses on the fate of metformin, its transformations in the presence of environmental minerals, and the toxicological impact on human cell lines. Experiments with humic acid and mineral-based photocatalysts (anatase, rutile, and amorphous titania) will uncover their role and critical insights. Prior studies have primarily focused on degradation using heterogeneous catalytic surfaces. In this work, we attempted to show that naturally occurring minerals could drive the formation of distinct degradation products of metformin in the environment. The surfaces of these minerals are likely to influence degradation rates and reaction mechanisms.

## 2. Materials and Methods

### 2.1. Materials

Metformin hydrochloride used in this study was a certified reference material from Sigma Aldrich. Anatase and rutile were sourced from the Source Clay Repository in Washington County, Georgia, and functioned as proxy environmental minerals in this study. Humic acid (Beantown Chemical, Hudson, NH, USA) was the representative organic matter, while amorphous titania was synthesized using titanium isopropoxide (Sigma Aldrich, St. Louis, MO, USA, ≥97% purity). For HPLC analysis, the solvent used was HPLC-grade methanol with ≥99.9% purity (VWR), while separation was best achieved with an ammonium acetate buffer (Sigma Aldrich, 99.99% purity).

HepG2 and HEK 293T cell lines were obtained from the American Type Culture Collection (ATCC) for cell viability assays. DMEM with L-glutamine was used as the growth medium supplied by Quality Biological. It was supplemented with Fetal Bovine Serum (Avantor, Radnor Township, PA, USA, ≥99%). The cells were treated with trypsin-EDTA solution (0.25%, 1X, VWR) for detachment. DMSO (ACS grade, VWR, West Chester, PA, USA) was used to prepare the assay reagents. Phenyl arsine oxide, PAO, ≥97%, by Spectral Chemicals, was used as a positive control.

### 2.2. Characterization of the Particles

Scanning electron microscopy (SEM) was used to determine the size and shape of the mineral particles. Images were captured at different magnifications and angles to determine the length and width. Approximately 250 particles for each mineral type were measured using ImageJ (Version 1.53e) software. Our previous studies explained the detailed protocols for SEM imaging and particle analysis methods [17,18,19]. A Nicolet IS50 spectrophotometer (Thermo Fischer Scientific, Waltham, MA, USA) with a mercury–cadmium–telluride (MCT) detector and Ge ATR element was utilized to obtain attenuated total reflectance-Fourier transform infrared (ATR-FTIR) spectra. Minerals were scanned at 4000 to 600 cm^−1^ range. Catalysts were prepared in water, sonicated, spread on the crystal, and left to dry before scanning.

### 2.3. Batch Reactor Studies

The degradation of metformin was carried out in custom-built glass reactors with a capacity of 100 mL, equipped with a water jacket to maintain the temperature. The reactor features a removable air-tight cap with a quartz window (12.5 cm^2^) for the entry of light. Then, 1 mM metformin hydrochloride (16.56 mg in 100 mL) solution was taken along with 0.1 g/L of minerals. Temperature was maintained at 25 °C, and samples were agitated using a magnetic stir bar to prevent precipitation. An oxygenated environment was created by bubbling oxygen for 5 min. A high drug concentration was loaded for low sensitivity in the toxicological assay. Experiments were conducted in both dark and light conditions. A 150 W xenon lamp (Newport Corporation, Irvine, CA, USA) with an Air Mass 1.5 G filter delivering a solar flux of ~105 mW/cm^2^ was used for solar simulations. The total reaction was carried out for 10 days, and samples were collected every 12 h (only on the first day at 0, 1, 3, 6, 9, and 12 h). Aliquots were drawn by a disposable syringe attached to 12 cm Teflon tubing. The samples were filtered with a 0.2 µm PTFE filter (Cole-Parmer, Vernon Hills, IL, USA) and kept in 2 mL collecting tubes. Each condition and catalyst were used in triplicate.

HPLC analysis was performed using a ZORBAX Eclipse Plus C18 (4.6 × 150 mm, 5 µm) reversed-phase column. A calibration curve was prepared using the standard metformin solutions. Degraded samples were thawed and analyzed in isocratic mode, with the mobile phase comprising 95% water and 5% methanol at a flow rate of 0.3 mL/min. Both mobile phases were buffered with 5 mM ammonium acetate, and the UV wavelength was set at 234 nm. The peak area from HPLC chromatograms was utilized to determine the concentration of metformin and normalized with respect to dark blank samples. Each data point was plotted to yield a decay curve for each experimental condition.

Degraded filtered samples were analyzed at the end of each batch reactor experiment to detect the transformed compounds using liquid chromatography–mass spectrometry (LC-MS). The analyses were performed using an Agilent 1290 LC coupled with an Agilent 6538 quadrupole time-of-flight (Q-TOF) mass spectrometer (Agilent Technologies, Santa Clara, CA, USA) at the Mass Spectrometry Facility of Montana State University. LC was carried out on a Kinetix 2.6 μm C18 column with dimensions of 2.1 × 150 mm (Phenomenex). The mobile phase consisted of solvent A: 0.1% formic acid in water and solvent B: 0.1% formic acid in acetonitrile. Samples were run at an elution rate of 0.1 mL/min with 99% of A. LC eluent was introduced to a positive electrospray ionization source.

### 2.4. Toxicological Assay

HEK 293 T and HepG2 cells were subsequently representative of kidney and liver cells. Cells were cultured in a humidified incubator using Dulbecco’s Modified Eagle Medium supplemented with Fetal Bovine Serum at 37 °C and 5% CO_2_. Log phase cells were treated with trypsin and mixed with media to prepare a cell suspension for plating. Cells were seeded in a 96-well plate at a concentration of 2000 cells/ well and allowed to adhere overnight. After 24 h, cells were treated with positive control, negative control, and varying concentrations of metformin or its final degraded solutions. Media were used as a negative control, and 100 μg/mL PAO was used as a positive control. Treated cells were allowed to grow for 48 h.

After 48 h, 20 µL of 3-(4,5-dimethylthiazol-2-yl)-2,5-diphenyltetrazolium bromide (MTT, Sigma-Aldrich, St. Louis, MO, USA) solution was added to each well, and again, the plates were incubated for 2 h. The supernatants were removed, and 100 µL of DMSO was added to dissolve the crystal violet formazan crystals. Absorbance was measured at 570 nm using a Coulter microplate reader. Data were expressed as % mean ± SD of treated/control (i.e., metformin or its photodegradation products) values obtained from three biological replicates. Two-way ANOVA was conducted at the 95% confidence level to determine the significant differences among all treatment groups. Unless otherwise mentioned, statistical significance was defined as *p* ≤ 0.05.

## 3. Results and Discussion

### 3.1. Characterization of Mineral Particles

**Figure 1:**
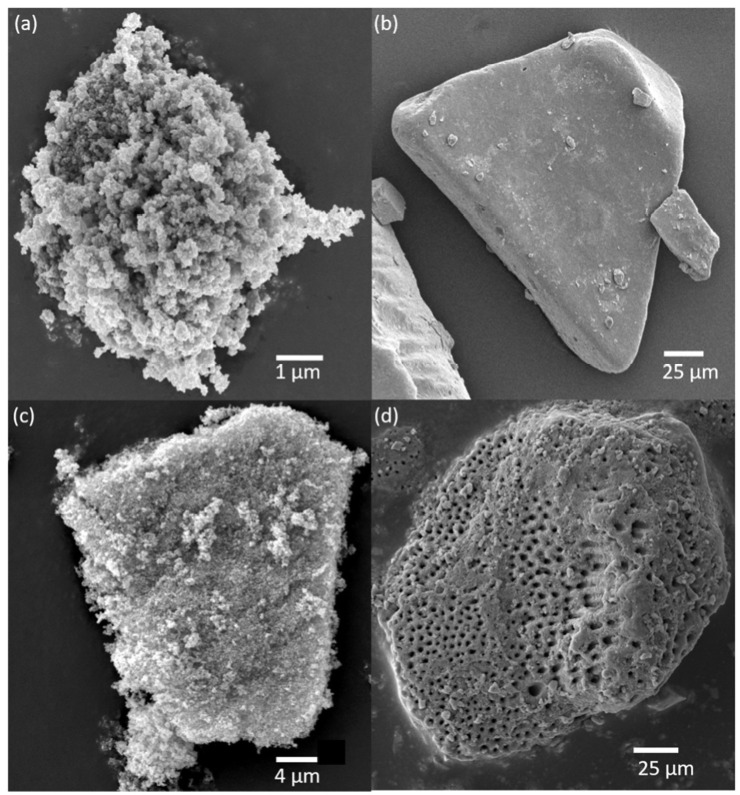
SEM images of (**a**) anatase, (**b**) humic acid, (**c**) rutile, and (**d**) amorphous titania.

Scanning electron microscopy was performed to reveal the crucial morphological characteristics of the mineral particles. Images were collected to examine the size and shape of these particles. Imaging was conducted at varying magnifications and different angles for comprehensive visualization of the particles. The length and width of approximately 250 particles were determined using the ImageJ (Version 1.53e) software. SEM images of the minerals are shown in Figure 1, and particle size distribution is presented in Figure S1.

Anatase and rutile are two crystalline forms of titania. Anatase features fine crystalline structures, while rutile particles are significantly larger and coarser than those of anatase. Amorphous titania reports the smallest particle sizes, which aligns with its lack of distinct crystallinity. In contrast, humic acid particles are the largest and exhibit a wide range of size distributions, reflecting their complex and irregular structures. These particle size differences can have a crucial impact on the degradation of metformin. Smaller particles like anatase and amorphous titania may have larger surface areas, enhancing adsorption and subsequent degradation. On the other hand, coarser minerals have a lesser surface area, which may result in less degradation.

**Figure 2:**
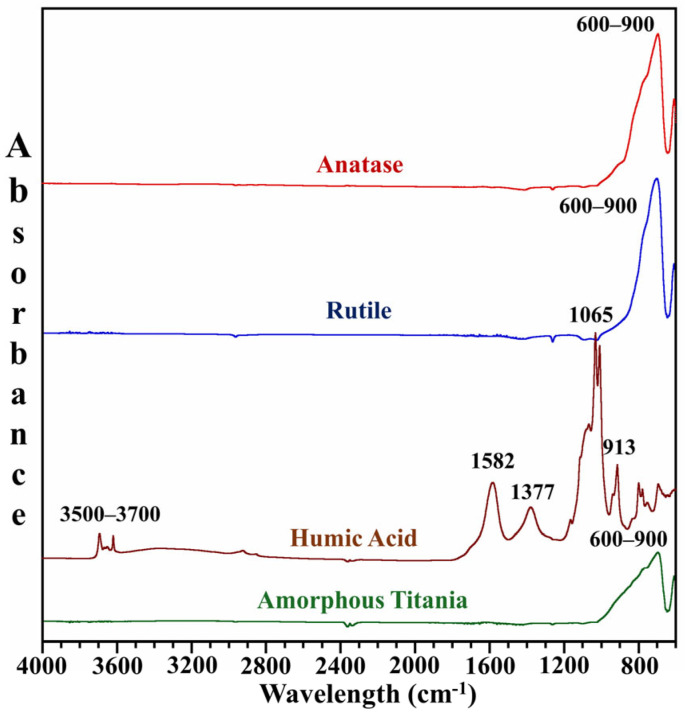
FTIR spectra of anatase, rutile, humic acid, and amorphous titania.

ATR-FTIR was performed to comprehend the surface functional groups of the four mineral particles. Representative FTIR spectra are shown in Figure 2. Anatase, rutile, and amorphous titania are three forms of TiO_2_ that exhibit a broad absorption band in the 600–900 cm^−1^ [20]. This band can be attributed to the stretching vibrations of Ti-O-Ti, which are characteristic of TiO_2_ phases. Despite their differences in crystallinity and surface characteristics, three forms of TiO_2_ showed similar backbones. These spectral features confirm the synthesis of amorphous titania and the integrity of the three photocatalysts.

Humic acid is a type of complex organic matter with a composition that varies widely, as reflected in its spectrum. The peaks between 3500 and 3700 cm^−1^ correspond to O-H or N-H stretching vibrations. These can be attributed to carboxylic acid groups, phenols, alcohols, amides, or amines, indicating the hydrophilic nature of humic acid and its interaction with polar molecules. A sharp peak at 1582 cm^−1^ represents the stretching vibrations of C=O for carboxylate functional groups or C=C in aromatic rings or alkenes. This suggests that aromatic and unsaturated components contribute significantly to the structure of humic acid. The peak around 1377 cm^−1^ can be associated with the stretching of phenolic O-H, the stretching of C-O in alcohols, or the vibrations of phenols and methyl groups. Additionally, spectral bands at 1065 cm^−1^ and 1030 cm^−1^ indicate the presence of aliphatic hydrocarbons and carbohydrate-like molecules in humic acid, respectively. The peak at 1065 cm^−1^ corresponds to the C−OH stretching of aliphatic compounds, while 1030 cm^−1^ relates to the C−O stretching of polysaccharide-associated substances. Finally, the peak at 913 cm^−1^ represents distinctive fingerprint bands of aromatic compounds [21,22,23], further emphasizing the role of aromatics in the molecular composition of humic acid. The abundance of these functional groups suggests they may play a crucial role in the degradation of metformin hydrochloride, consequently impacting the environment.

### 3.2. Degradation Studies

The degradation studies of metformin were performed in the presence of anatase, rutile, amorphous titania, and humic acid. Experimentation with amorphous titania was conducted to evaluate the crystalline effect on degradation. Experiments were carried out under dark and irradiated conditions to simulate the nighttime and daytime processes. Our results highlight no significant degradation under dark conditions over the 240 h. The decay curves under dark conditions are provided in the Supporting Information (Figure S2).

**Figure 3:**
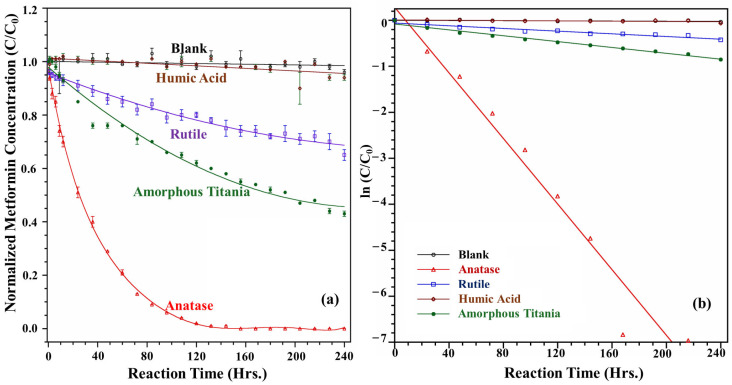
Degradation and kinetics study of metformin. (**a**) Degradation of metformin in solar conditions. (**b**) Kinetics of metformin decay at air mass filter 1.5 G.

Under irradiated conditions (Figure 3a), no degradation was observed in the absence of mineral particles. The humic acid concentration decreased slightly, and HPLC chromatograms did not show any peaks indicative of degradation. The presence of functional groups on the humic acid surface was insufficient to break down metformin. Although this outcome may seem surprising, it parallels how metformin is metabolized in the human body. Metformin remains unchanged and is excreted in the urine, even after exposure to various enzymes [3]. Additionally, the aromatic and oxygen-containing functional groups make humic acid chromophoric, which means they can absorb UV/Vis light to excite and transfer energy or electrons to oxygen to generate reactive oxygen species. However, the quantum yield of strong oxidants produced by humic acid at longer wavelengths is much lower than that of •OH generated from TiO_2_. In the absence of any photocatalyst, humic acid acts as a weak photosensitizer. They produce mild singlet oxygen (^1^O_2_) or undergo self-sensitized redox reactions, rather than aggressively oxidizing pollutants via •OH [24]. Furthermore, humic acid can behave as a radical scavenger or quencher by binding to photocatalyst surfaces and consuming photogenerated holes (h^+^), thereby inhibiting •OH production [25]. For this reason, despite having numerous useful functional groups, the net photoreactivity of humic acid was limited. Anatase demonstrated the highest degradation of the photocatalysts tested, completely breaking down within 156 h. Representative chromatograms for the metformin degradation by anatase under irradiated conditions are provided in the Supporting Information section (Figure S3). In contrast, rutile only degraded by more than a third over the course of 10 days, which is significantly slower than anatase.

The degradation of metformin can be described using a pseudo-first-order kinetic model, represented by the following equation:

ln (C/C_0_) = −kt (1)

In equation (1), C_0_ and C are the metformin concentrations at the initial time and time t, respectively. The rate constants, correlation coefficients, and half-lives for each solar condition are summarized in Table 1. Kinetic data are illustrated in the graph provided in Figure 3b. After fitting the data to the pseudo-first-order model, minor deviations were observed. These deviations may be attributed to various heterogeneous processes, such as forming adsorption complexes, equilibrium reactions between adsorbed and free species, and desorption processes [26].

Anatase demonstrated a significantly high rate constant (k = 0.0356 h^−1^) with a half-life of 19.5 h, indicating its superior photocatalytic activity. In contrast, rutile degraded metformin at a slower rate, with a rate constant of 0.0015 h^−1^ and a longer half-life of 462 h. The degradation with amorphous titania was intermediate, exhibiting a half-life of 216 h. All the equations showed linearity with a high R² value, which supports their fitting to a pseudo-first-order kinetic model.

Given that anatase, rutile, and amorphous titania are three forms of TiO_2_, they can absorb photons with energy equal to or greater than their bandgap, and electrons in the valence band are excited to the conduction band. This process generates electron–hole pairs (e^−^ and h^+^), which are essential for photocatalysis. The generated electrons and holes can be trapped at defective sites or surface states, recombined, or transferred to the TiO_2_ surface to initiate redox reactions [27,28]. For TiO_2_, trapping increases the lifetime of the charged carriers, while recombination reduces the efficiency of photocatalysts. The holes are transferred to the surface and participate in redox reactions. Electrons reduce oxygen molecules to form reactive oxygen species (ROS), while holes oxidize water to produce oxidized products such as hydroxyl radicals (•OH) [29,30]. Based on the prior studies, it is well established that these oxidized products and ROS play a dominant role in degradation under aerated photocatalytic conditions [26A]. Photocatalytic degradation often benefits from the adsorption of molecules onto catalyst surfaces [31]. In our study, negligible adsorption of metformin was observed in the dark control conditions, which can be attributed to its polar and hydrophilic nature that limits its interaction with mineral surfaces. Nonetheless, the formation of ROS and •OH radicals on the illuminated surface of TiO_2_ diffuses into solution and degrades metformin even in the absence of significant surface adsorption. The smaller particle sizes of anatase and amorphous titania increase surface area, which may facilitate slightly higher adsorption and catalytic interaction than rutile to increase degradation performance [32].

The degradation kinetics of anatase and rutile can be attributed to the superior photocatalytic properties of anatase compared to those of rutile. While there is no universally accepted explanation for why anatase is a better photocatalyst, one acknowledged reason is that anatase has a smaller indirect bandgap compared to its direct bandgap. In contrast, rutile’s direct and indirect band gaps are nearly identical. Distinct indirect band gaps in anatase lead to longer charge carrier lifetimes. This prolonged existence of electron–hole pairs in anatase is advantageous for surface reactions [33]. Also, anatase photo-absorbs and photo-desorbs oxygen more effectively and recombines fewer electron–hole pairs. Its hydroxyl radical generation rate is higher, crucial for its photocatalytic reactions [34]. On the other hand, the smaller distance between Ti-Ti on a rutile surface forms structures such as Ti-OO-Ti, leading to the formation of O_2_ and reducing its reactivity [35]. Although amorphous titania is not commonly found in nature, batch reactor studies have been conducted to assess the impact of crystallinity. The experiments showed that amorphous titania effectively degraded metformin, highlighting the importance of photocatalysts in degradation processes. Amorphous titania lacks the long-range atomic order of crystalline phases and contains numerous defect sites, which act as charge recombination centers. Due to this recombination, amorphous titania shows lower photocatalytic activity. The ordered lattice and proper band structure of anatase facilitate reactive charge carriers, whereas disorder of amorphous TiO_2_ impedes photocatalytic oxidation [36].

**Figure 4:**
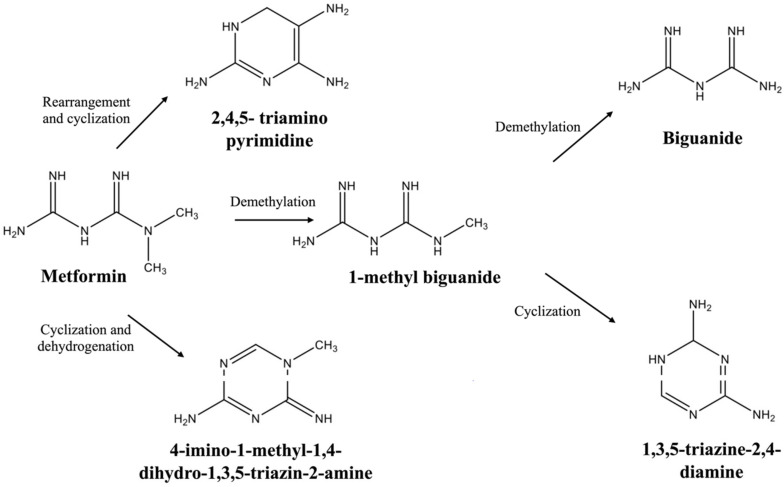
Proposed degradation mechanism of metformin.

The resulting structures of degraded metformin were identified using liquid chromatography coupled with a quadrupole time-of-flight (Q-TOF) mass spectrometer. Here, we utilized PubChem, SpectraBase, and the NIST Mass Spectral Library as references to identify and verify the major peaks and, consequently, the degraded products. These databases provide a platform for cross-referencing established molecular mass and fragmentation data. Additionally, previously published fragmentation patterns helped confirm the structures [12,14]. Using the high-resolution mass spectra and their fragmentation patterns, we detected five major transformation products: 4-Imino-1-methyl-1,4-dihydro-1,3,5-triazin-2-amine, 1,3,5-Triazine-2,4-diamine, 2,4,5-triaminopyrimidine, biguanide, and 1-methyl biguanide. Fragmentation patterns for these compounds are included in the Supporting Information section (Figure S4). It is important to note that all three degradation surfaces—anatase, rutile, and amorphous titania—produced the same transformation products, although their relative abundances varied. Most of these compounds are commonly recognized as impurities or as degradation products of metformin. The data generated from LC-MS serve as a foundation for proposing potential degradation mechanisms of metformin (Figure 4) and provide an opportunity to evaluate the toxicity of these degraded compounds.

An oxygenated environment enriches the dissolved oxygen, ensuring an oxygen-saturated environment. O_2_ acts as an electron acceptor and promotes the formation of reactive oxygen species (ROS), which can attack metformin and lead to its transformation. The dominance of •OH has been shown explicitly for the degradation of various organic molecules, while other species like superoxide (O_2_^−^•) or singlet oxygen may form, they typically play secondary roles [37]. Trapping experiments performed with organic compounds also confirmed the contribution of •OH radicals as a major contributor [38]. When these free radicals target the nitrogen atom in the two methylene groups of metformin, the compound undergoes demethylation, forming 1-methyl biguanide. This intermediate can then be further demethylated to produce biguanide. In another pathway, 1-methyl biguanide can become excited and undergo cyclization, forming 1,3,5-triazine-2,4-diamine. Dehydrogenation and cyclization can also yield another common metformin degradation product, 4-imino-1-methyl-1,4-dihydro-1,3,5-triazin-2-amine [39]. Moreover, metformin can undergo cyclization in a different pathway while hydrogen atoms are removed, forming 2,4,5-trimethylpyrimidine. Reactive oxygen species play a crucial role in forming degraded compounds, which complicates the understanding of their toxicological implication.

These mechanisms also comply when analyzed in conjunction with relative abundance data. Relative abundance is listed in the Supporting Information (Table S1). Since anatase completely degraded metformin, 2,4,5-triaminopyrimidine and 4-imino-1-methyl-1,4-dihydro-1,3,5-triazin-2-amine were the most abundant compounds in anatase degraded solutions. The amount of 1-methyl biguanide is the least in anatase-degraded solutions, indicating sufficient time to convert into biguanide and 1,3,5-triazine-2,4-diamine. In contrast, the abundance of these compounds is the lowest in rutile solutions, as degradation is less, and those metformin converted into 1-methyl biguanide cannot transform into other compounds. Thus, the relative abundance data confirm that metformin forms 1-methyl biguanide and then converts into biguanide and 1,3,5-triazine-2,4-diamine.

### 3.3. Cell Viability Assay

Given that metformin degrades in the presence of a photocatalytic mineral and solar flux, the degraded mixtures of the three photocatalysts, after 240 h of degradation, were used to evaluate their acute toxicity levels via a cell viability assay while comparing with the respective parent compounds. These in vitro studies were performed using two human cell lines: HEK 293T and HepG2, which are representative of kidney and liver cells, respectively. Three negative controls were required to prepare for three different concentrations, as water was the solvent of the mixtures, and PAO was used as a positive control, representing the condition of non-viable cells in the entire cell population. All the data were normalized with negative values and reported as % cell viability ± standard deviation.

**Figure 5:**
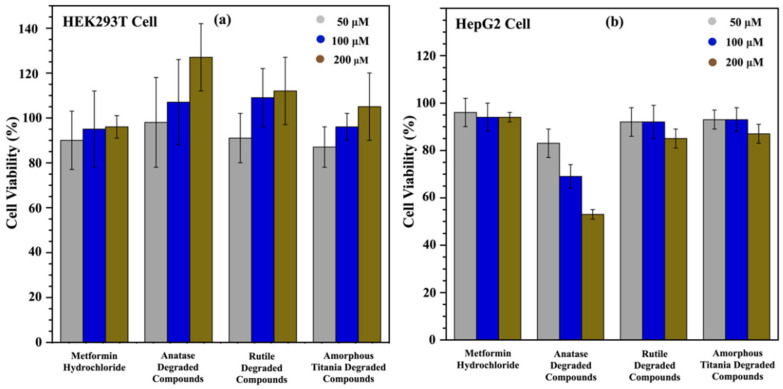
Investigation of metformin hydrochloride and its photodegraded mixtures on the proliferation of human cell lines (**a**) HEK 293T (kidney) cell line, (**b**) HepG2 (liver) cell line.

Three different concentrations were tested for metformin and its degraded compounds: 50 µM, 100 µM, and 200 µM. For kidney cell lines, the viability of cells increased with higher concentrations in most cases (Figure 5a). However, the viability of the cells in response to metformin was not dependent on the concentration within this range. This could be due to the small stimulatory effect known as a hormetic effect, where low concentrations cause a modest stimulation followed by inhibition at higher concentrations [40]. It can be concluded that 200 µM is insufficient to cause toxicity with metformin. The observed increase in the viability of cells with higher concentrations could be an artifact of the assay or explained by metformin’s functional activity; since this drug works on cellular metabolism, the increased viability may be correlated with the increment of drug doses [41]. Further studies with higher concentrations and individual degraded compounds could provide a clearer understanding of metformin’s nephrotoxicity.

Unlike HEK 293T cells, HepG2 cells showed decreased cell viability with increased concentrations of anatase within the error. This does seem to be supported by the data (Figure 5b). Although the data for metformin, rutile solution, and amorphous titania degraded compounds are not statistically significant, they show a downward trend, indicating that they may affect cell viability at higher concentrations. A two-factor ANOVA was performed to check statistical significance. Overall, it was observed that the variation due to concentration was not significant (*p* = 0.14), whereas treatment with different minerals showed a substantial difference (*p* = 0.01). Tukey’s pairwise comparison tests were performed with a 95% confidence level, and the results showed that anatase exhibited significantly higher toxicity than any other mixture. The degradation studies found that anatase solutions contained no metformin, only a mixture of degraded compounds. Thus, our results suggest that the degraded products could be more toxic than the parent metformin. Even though we did not observe a significant difference at lower concentrations among the mineral treatments, the solutions of different minerals were significantly different (except rutile and amorphous titania) at higher concentrations. This indicates the potential toxicity of metformin and its degraded compounds at higher concentrations. Further studies on their toxicity profiles are proposed for a comprehensive sketch of the human health effects of these secondary compounds.

## 4. Conclusions

This study advances our understanding of the environmental degradation of metformin hydrochloride and its toxicological impacts. Leveraging natural minerals, we explored how the intrinsic properties of these catalysts influence the breakdown of this pharmaceutical compound. Our study revealed that photocatalysts are highly efficient in breaking down metformin under irradiated conditions. Anatase, a better photocatalyst than rutile, particularly breaks more than half of the metformin daily. Experimentation with amorphous titania underscores the photocatalytic activity over the crystallinity effect. Interestingly, despite having numerous functional groups on the surface, humic acid showed minimal impact on degradation.

The degraded compounds of metformin, elucidated through LC-MS, were analyzed and compared with the MS spectra databases. The formed products for all the photocatalysts were the same, except for their abundance. Each mineral has a distinct degradation efficacy and influences the distribution of metformin’s degradation products. While anatase has the fastest kinetics, consistent with its superior photocatalytic activity, the lower activity of rutile resulted in the accumulation of early-stage intermediates like 1-methylbiguanide. Degradation with amorphous titania, despite lacking crystallinity, further highlights the role of photocatalyst properties in metformin degradation.

In contrast, humic acid showed a minimal degradative effect, underscoring that natural organic matter alone is ineffective in deleting metformin. These compounds, mostly previously known as the impurities of metformin and reactive oxygen species, may play a vital role in forming these products through demethylation, cyclization, and dehydrogenation. However, most of the compounds in this experiment were proposed based on the available mass spectroscopy data, the validity of which could have been further ensured by other techniques, such as nuclear magnetic resonance (NMR) spectroscopy. Finally, a colorimetric assay was accomplished to explore acute nephrotoxicity and hepatotoxicity, and results imply that metformin has a more pronounced effect on liver cell viability than kidney cells. This necessitates the evaluation of the toxicity of the parent compound with individual degraded compounds of metformin. Comprehensive toxicological studies could provide detailed information to understand the toxicological impact. In addition, advanced molecular and cellular analyses will elucidate pathways for cellular damage and identify the responsible entity for cellular toxicity. Additionally, evaluating the effect on other organisms, such as gut microbiota or marine organisms, or scaling up these findings in wastewater treatment systems will be vital for practical applications. Collaboration between chemists, ecologists, and engineers can bridge these gaps and offer sustainable solutions to the persistent challenge of pharmaceutical pollution.

## Figures and Tables

**Table 1:** Rate constants, correlation coefficient, and half-life of metformin hydrochloride at solar conditions on different mineral surfaces.

Surface	Rate Constant (h^−1^)	R^2^	Half-Life (hours)
Anatase	0.0356	0.965	19.5
Rutile	0.0015	0.930	462
Amorphous Titania	0.0032	0.981	216

## Data Availability

Data will be made available upon request.

## Supplementary Materials

The following supporting information can be downloaded at: https://www.mdpi.com/article/10.3390/toxics13050407/s1, Figure S1: Particle size distribution of anatase, humic acid, rutile and amorphous titania; Figure S2: Comparison of metformin degradation with different minerals in dark conditions; Figure S3: Degradation of metformin by anatase in solar condition at different time point; Table S1: Relative abundance (peak area) of degraded compounds of metformin; Figure S4: Fragmentation pattern of metformin and its degraded products.
