# Supplementary material for: Role of Environmental Photocatalysts and Organic Matter on the Degradation and Toxicity of Metformin Hydrochloride
Source: Toxics. 2025 May 17;13(5):407. doi: 10.3390/toxics13050407 (PMC12115835; doi:10.3390/toxics13050407)
Supplement: Supplementary file 1 [file toxics-13-00407-s001.zip › toxics-3614721-supplementary.pdf]

Supporting Information

Role of Environmental Photocatalysts and Organic Matter on the Degradation & Toxicity of Metformin Hydrochloride

Rifat Khan, Jaqueline Regalado, Malsha Indeewari Kanaththage, Gayan Rubasinghege*

Department of Chemistry, New Mexico Institute of Mining & Technology, 801 Leroy Place, Socorro, NM 87801, USA

*Correspondence: gayan.rubasinghege@nmt.edu

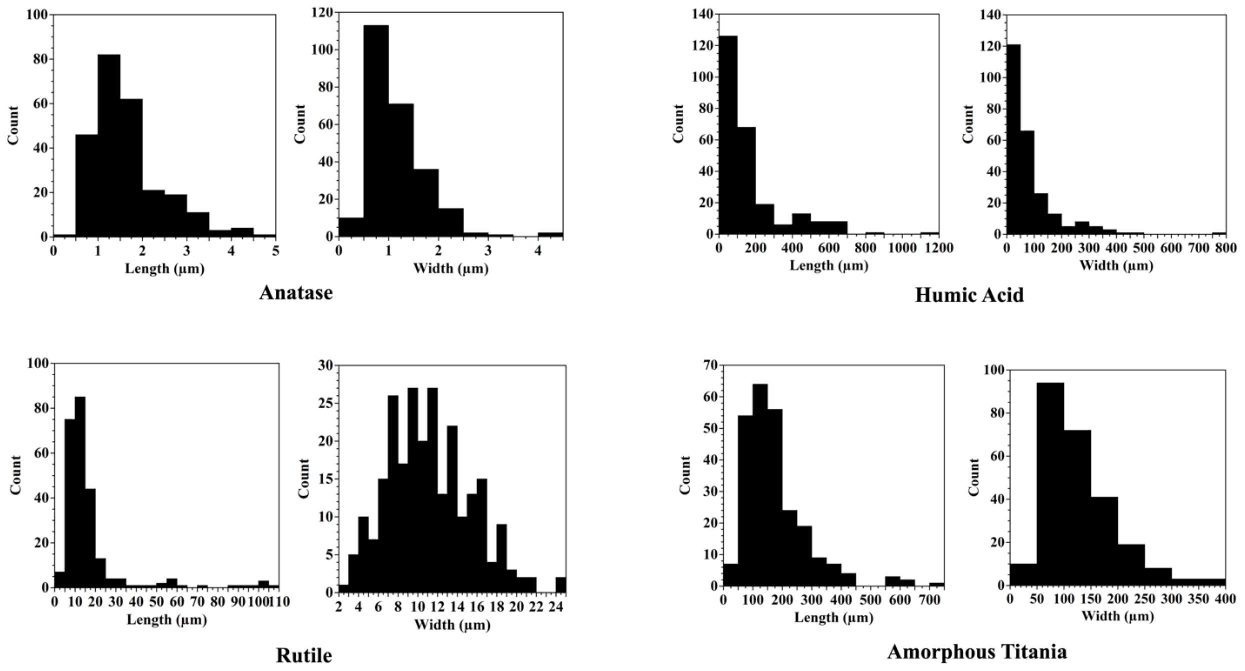

Figure S1. Particle size distribution of anatase, humic acid, rutile and amorphous titania

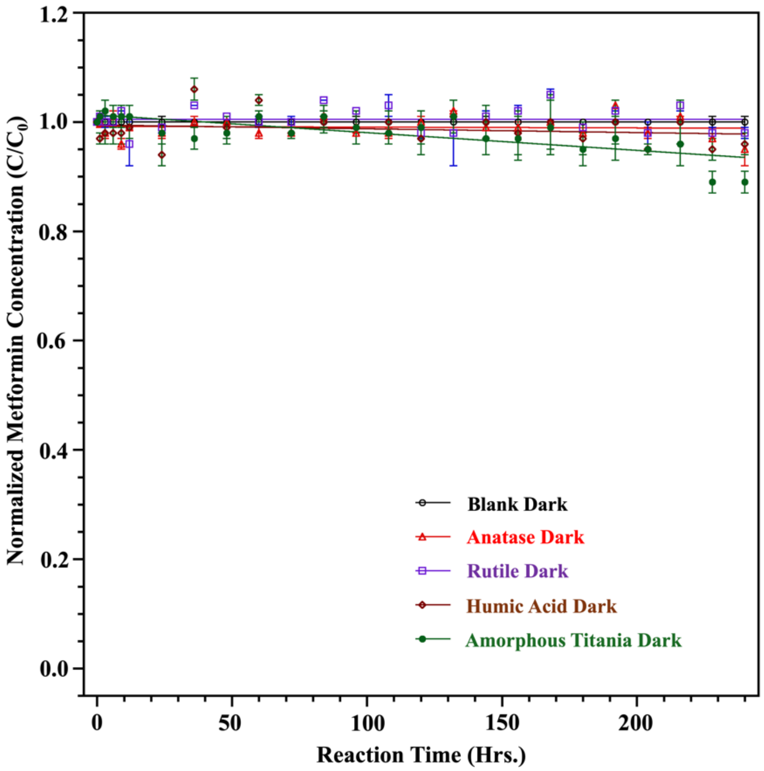

Figure S2. Comparison of metformin degradation with different minerals in dark conditions

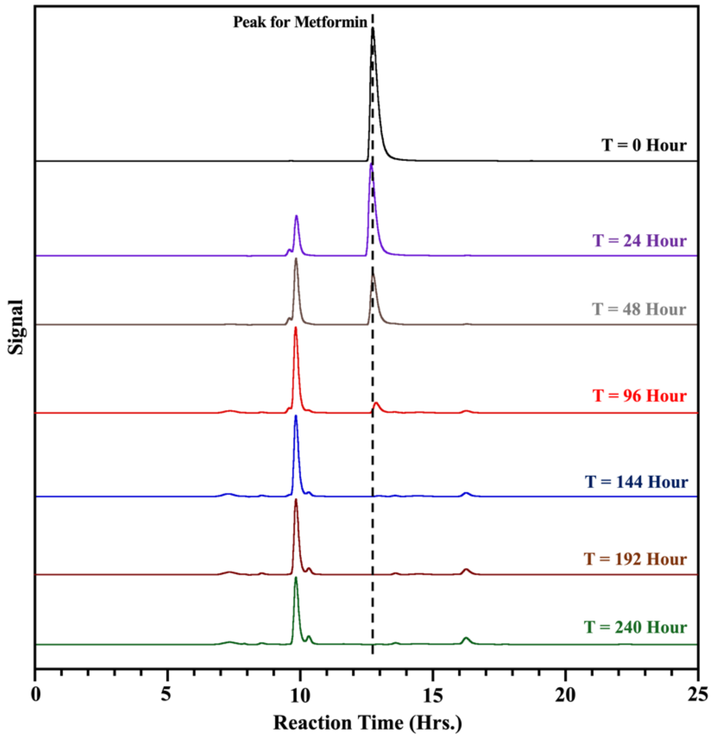

Figure S3. Degradation of metformin by anatase in solar condition at different time point

Table S1. Relative abundance (peak area) of degraded compounds of metformin

Compound Name	Molecular mass (g)	Retention Time (s)	In anatase degraded solution	In rutile degraded solution	In amorphous titania degraded solution
2,4,5-triaminopyrimidine	125.0695	3.93	14,230,543	64,723,739	10,648,052
4-imino-1-methyl-1,4-dihydro-1,3,5 triazin-2-amine	125.0695	5.03	27,128,591	40,824,469	4,302,677
1,3,5-triazine-2,4-diamine	111.0538	4.07	954,853	34,863,785	1,286,501
1-methyl biguanide	115.0853	3.62	2,678,517	268,187	1,274,906
Biguanide	101.0697	3.53	82,852	671,921	313,805

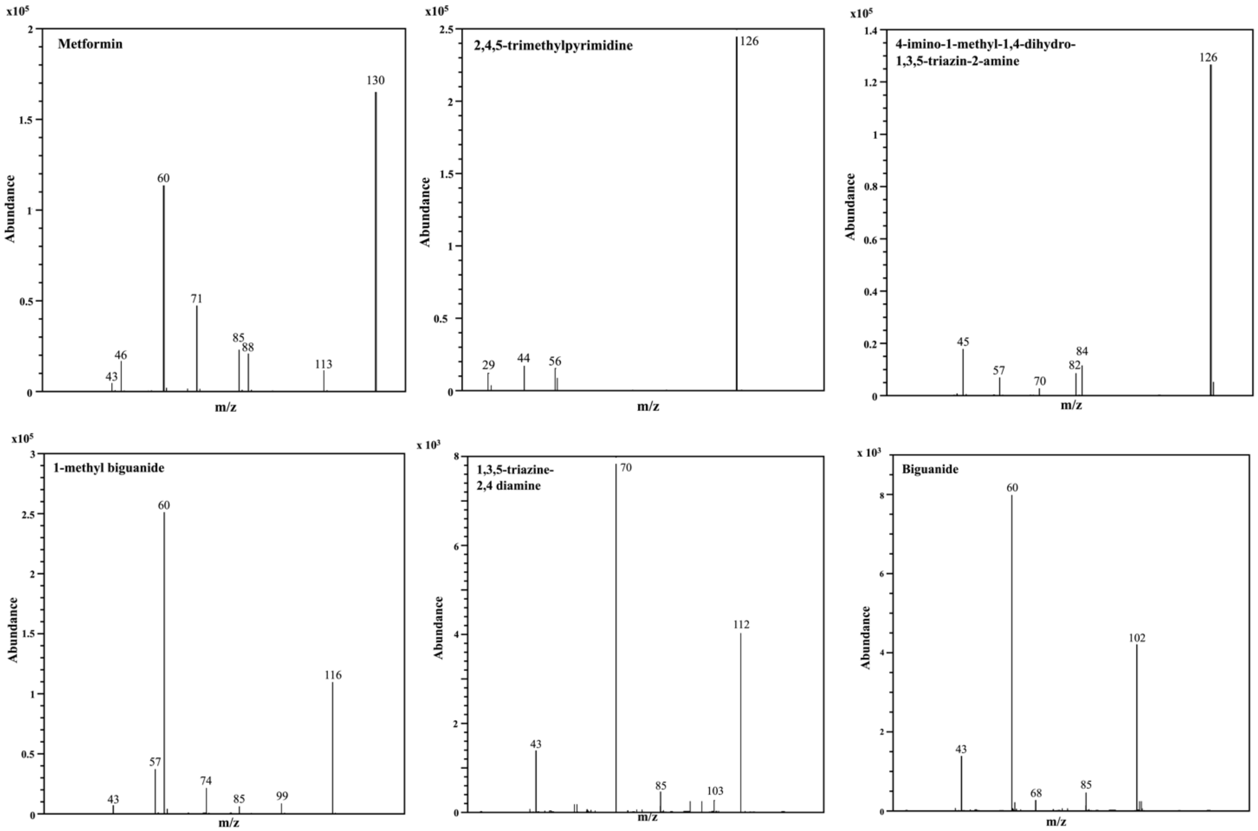

Figure S4. Fragmentation pattern of metformin and its degraded products
